# Loss of Pfizer (BNT162b2) Vaccine-Induced Antibody Responses against the SARS-CoV-2 Omicron Variant in Adolescents and Adults

**DOI:** 10.1128/jvi.00582-22

**Published:** 2022-08-17

**Authors:** Sneh Lata Gupta, Grace Mantus, Kelly E. Manning, Madison Ellis, Mit Patel, Caroline Rose Ciric, Austin Lu, Jackson S. Turner, Jane A. O’Halloran, Rachel M. Presti, Devyani Jaideep Joshi, Ali H. Ellebedy, Evan J. Anderson, Christina A. Rostad, Mehul S. Suthar, Jens Wrammert

**Affiliations:** a Division of Infectious Diseases, Department of Pediatrics, School of Medicine, Emory University, Atlanta, Georgia, USA; b Emory Vaccine Center, Emory University grid.189967.8grid.471395.dgrid.189967.8 School of Medicine, Atlanta, Georgia, USA; c Centers for Childhood Infections and Vaccines, Children's Healthcare of Atlanta, Atlanta, Georgia, USA; d Department of Microbiology and Immunology, Emory University grid.189967.8grid.471395.dgrid.189967.8 , Atlanta, Georgia, USA; e Department of Medicine, Emory University grid.189967.8grid.471395.dgrid.189967.8 School of Medicine, Atlanta, Georgia, USA; f National Primate Research Center, Atlanta, Georgia, USA; g Department of Pathology and Immunology, Washington University School of Medicine, St. Louis, Missouri, USA; h The Andrew M. and Jane M. Bursky Center for Human Immunology & Immunotherapy Programs, Washington University School of Medicine, St. Louis, Missouri, USA; i Center for Vaccines and Immunity to Microbial Pathogens, Washington University School of Medicine, St. Louis, Missouri, USA; j Division of Infectious Diseases, Department of Internal Medicine, Washington University School of Medicine, St. Louis, Missouri, USA; St. Jude Children's Research Hospital

**Keywords:** Pfizer-BioNTech (BNT162b2), antibody binding titers, variant of concerns (VOCs), adolescents, virus neutralization, seasonal beta coronavirus, COVID-19, Omicron (B.1.1.529), SARS-CoV-2

## Abstract

Emerging variants, especially the recent Omicron variant, and gaps in vaccine coverage threaten mRNA vaccine mediated protection against SARS-CoV-2. While children have been relatively spared by the ongoing pandemic, increasing case numbers and hospitalizations are now evident among children. Thus, it is essential to better understand the magnitude and breadth of vaccine-induced immunity in children against circulating viral variant of concerns (VOCs). Here, we compared the magnitude and breadth of humoral immune responses in adolescents and adults 1 month after the two-dose Pfizer (BNT162b2) vaccination. We found that adolescents (aged 11 to 16) demonstrated more robust binding antibody and neutralization responses against the wild-type SARS-CoV-2 virus spike protein contained in the vaccine compared to adults (aged 27 to 55). The quality of the antibody responses against VOCs in adolescents were very similar to adults, with modest changes in binding and neutralization of Beta, Gamma, and Delta variants. In comparison, a significant reduction of binding titers and a striking lack of neutralization was observed against the newly emerging Omicron variant for both adolescents and adults. Overall, our data show that a two-dose BNT162b2 vaccine series may be insufficient to protect against the Omicron variant.

**IMPORTANCE** While plasma binding and neutralizing antibody responses have been reported for cohorts of infected and vaccinated adults, much less is known about the vaccine-induced antibody responses to variants including Omicron in children. This illustrates the need to characterize vaccine efficacy in key vulnerable populations. A third (booster) dose of BNTb162b was approved for children 12 to 15 years of age by the Food and Drug Administration (FDA) on January 1, 2022, and pediatric clinical trials are under way to evaluate the safety, immunogenicity, and effectiveness of a third dose in younger children. Similarly, variant-specific booster doses and pan-coronavirus vaccines are areas of active research. Our data show adolescents mounted stronger humoral immune responses after vaccination than adults. It also highlights the need for future studies of antibody durability in adolescents and children as well as the need for future studies of booster vaccination and their efficacy against the Omicron variant.

## INTRODUCTION

According to the World Health Organization (WHO), COVID-19 cases from SARS-CoV-2 infection exceed 551 million with over 6.3 million deaths worldwide. As reported, people aged 65 and above are at higher risk of infection, while children 0 to 17 years of age have been reported to have fewer cases compared to adults (1, 2). Despite the smaller number of cases, children can still develop SARS-CoV-2 infection and symptoms and can spread the infection (3). Some children may also develop severe COVID-19 symptoms requiring hospitalization (4, 5) and potentially leading to serious long-term health effects. Furthermore, some children can develop a life-threatening condition known as multisystem inflammatory syndrome in children (MIS-C) (6, 7). Importantly, with the emergence of the Omicron variant, a significant increase in the number of SARS-CoV-2 infections and hospitalizations in children has been observed (8).

The Pfizer (BNT162b2) vaccine is a lipid nanoparticle-based mRNA vaccine that encodes full-length spike protein and is given at 30 μg/dose with a 21-day interval for adults (9). In the United States, the FDA has approved the use of Pfizer-BioNTech COVID-19 vaccine in children in a stepwise manner, with >16 years of age approved in December 2020, adolescents aged 12 to 16 approved in May 2021, and children aged 5 to 11 years approved in October 2021 (10, 11).

The continued emergence of new viral variant of concerns (VOCs) illustrates the need for ongoing evaluation of vaccine immunogenicity and efficacy. While vaccine induced immunity has been largely retained against emerging viral variants thus far, significant reduction in neutralizing potential has been observed, especially for the B.1.351 (Beta) VOC (12). Pfizer-vaccinated adults showed a 6- to 7-fold reduction in neutralization titers against B.1.617.1 and 3- to 4-fold reduction against the Delta variant (B.1.617.2) compared with the prototype emergent virus (WA1) (13). More recent studies with both COVID-19-infected individuals and fully BNT162b2-vaccinated individuals show significantly reduced neutralizing titers against the emerging Omicron variant compared with the original wild-type strain and to other VOCs (14, 15). In fact, most SARS-CoV-2 naive vaccine recipients failed to neutralize the Omicron 1 month after the second vaccine dose. Individuals receiving a third booster dose of the BNT162b2 at 6 months showed improved neutralizing titers against the Omicron as did previously SARS-CoV-2 infected vaccinees (16).

In the current study, we have compared the humoral immune response in two groups of vaccinees, an adolescent and an adult cohort. We recruited 15 adolescents, with a median age 13 (11–16), who had received two doses of Pfizer vaccine and analyzed their antibody binding response and neutralization titers 1 month after the second dose of vaccination. Their antibody response was compared with 18 adults, median age 35 (27 to 55), 1 month postvaccination. Our data showed adolescents exhibit greater vaccine induced antibody binding and neutralization titers compared with the adults. Additionally, both binding and neutralization titers displayed an equivalent decline against a panel of VOCs suggesting the functional antibody repertoire in adolescents and adults are similar. Strikingly, we found vaccine-induced immunity displayed minimal activity against the Omicron variant, in terms of binding and neutralization titers in adolescent donors, similar to adult donors. Overall, our data showed adolescents mount a more potent antibody response to vaccination, with a similar breadth of reactivity against VOCs compared with adults. Furthermore, we showed both cohorts display minimal neutralization activity against the novel Omicron variant.

## RESULTS

### Adolescent vaccinees mount a stronger antibody response to the Pfizer vaccine than adults.

To determine the magnitude of binding and neutralizing antibody responses in adult and adolescent vaccine recipients, we employed a combination of a multiplex Mesoscale Discovery (MSD) platform for binding analyses, and a live virus focus reduction neutralization (FRNT) assay for neutralization analysis. We recruited 15 adolescent participants, enrolled July to August 2021, with a median age of 13 years (range:11 to 16; 80% female), while the 18 adult participants were enrolled February to March 2021, having a median age of 35 years (range: 27 to 55; 44% female) (Table 1). Both groups received two doses of Pfizer (BNT162b2) vaccine and their antibody responses were analyzed 1 month after the second dose of vaccination. Pfizer vaccine is based on spike mRNA that includes the receptor binding domain (RBD) and N-Terminal Domain (NTD). Herein, we quantified the antibody binding titers using the SARS-CoV-2 MSD panel 1.

**TABLE 1:** Adolescents and adults demographic data participated in this study

Cohort	Total no.	Adolescents 15	Adults 18
Sex	Female (%)	12 (80%)	8 (44.4%)
	Male (%)	3 (20%)	10 (55.5%)
Median age (range)		13 (11 to 16)	35 (27 to 55)
Race	White (%)	6 (40%)	15 (83.3%)
	Asian (%)	5 (33.3%)	2 (11.1%)
	Others (%)	4 (26.6%)	1 (5.55%)
Ethnicity	Hispanic (%)	2 (13.3%)	1 (5.55%)
	Non-Hispanic (%)	13 (86.6%)	17 (94.44%)
Samples collected		July to August 2021	February to March 2021

**FIG 1:**
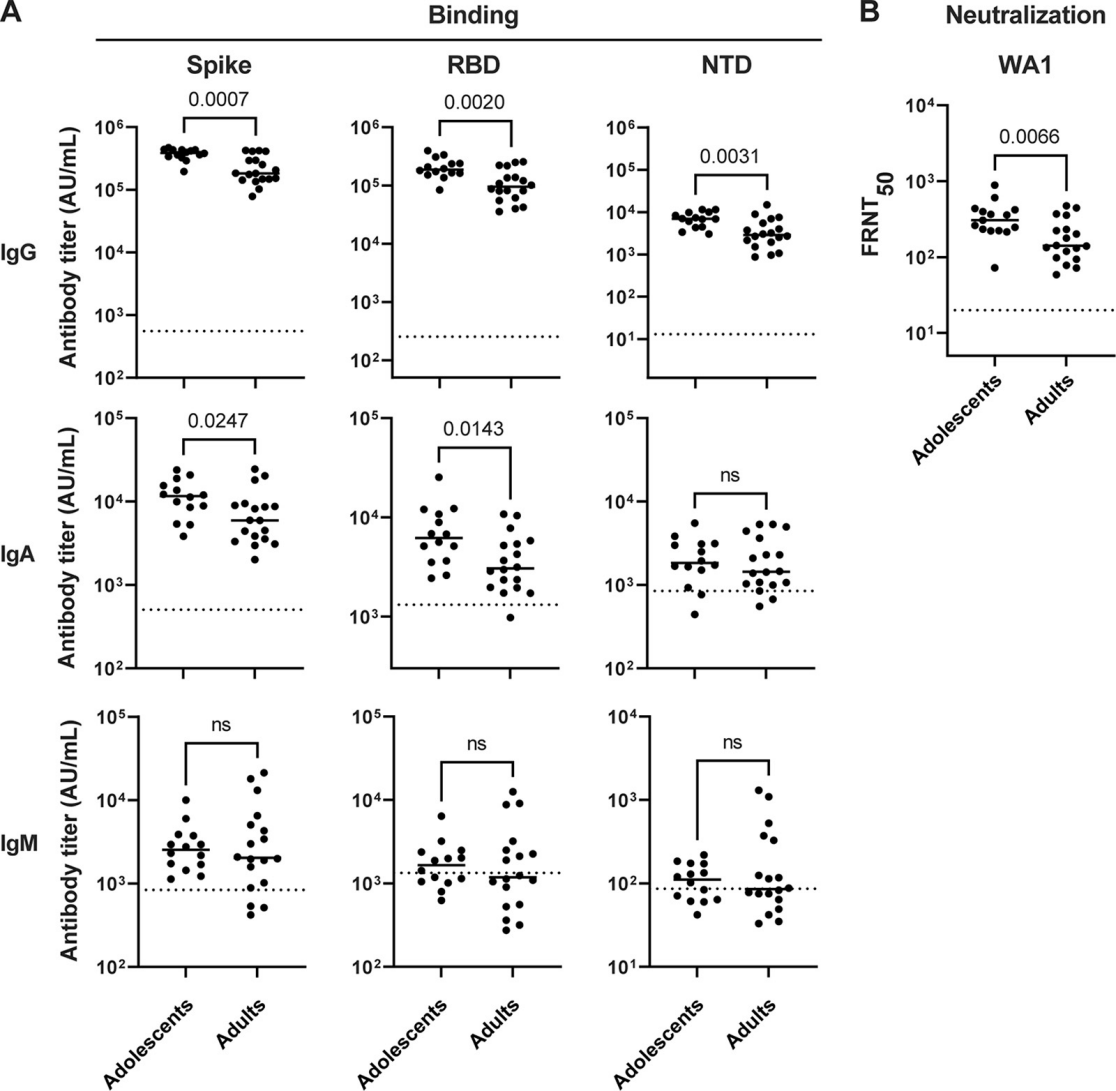
Vaccinated adolescents display higher binding and neutralization titers compared with adult donors. Plasma samples were tested in duplicate for IgG, IgA, and IgM antibody binding titers against (A) SARS-CoV2 Spike, RBD, and NTD comparing adolescents (*n* = 15) and adults (*n* = 18). Dotted line represents the limit of detection which is set by binding titers of prepandemic adult healthy controls (*n* = 8) calculating as (average +3 SD). (B) Both adolescent (*n* = 15) and adult (*n* = 18) plasma samples were analyzed for their ability to neutralize the SARS-CoV-2/USA/WA1/2020 virus strain. Dotted line represents the limit of detection at 1/20 (with undetectable titers assigned a value of half of lowest dilution = 1/10).

Adolescents showed significantly higher IgG binding titers for the full-length spike protein (*P* value = 0.0007), as well as the S1 RBD (*P* value = 0.0020), and NTD (*P* value = 0.0031) compared to adults (Fig. 1A). In adolescents, the Spike IgG binding titers had a median of 388,975 AU/mL (range 196,141 to 469,080) while in adults the median was 182,997 AU/mL (range 78,334 to 425,117). Comparing S-1 RBD binding titers between adolescents median 187,034 AU/mL (range 83,972 to 395,205) and adults median 94,459 AU/mL (range 35,589 to 25,4698) showed a similar difference. For the NTD domain, the antibody binding titers in adolescent had a median of 6,946 AU/mL (range 3,016 to 11,499) while in adults it was 2,868 (range 957 to 14,819). We also found that adolescents had higher IgA Spike and RBD binding titers compared to adults, while there was no difference in IgM binding titers at 1 month postvaccination compared to adults (Fig. 1A). To assess vaccine induced neutralization capacity in adults and adolescents, we utilized a live virus neutralization assay with WA1. Closely mirroring the binding data above, we found significantly higher (*P* value = 0.0066) neutralizing titers against WA1 in the adolescent cohort Geometric Mean Titer (GMT) 304 (range 72 to 884) than in adults GMT 163 (range 72 to 473) (Fig. 1B). Similar to prior findings, we observed a close correlation between SARS-COV-2 binding and neutralization titers (data not shown) (17).

**FIG 2:**
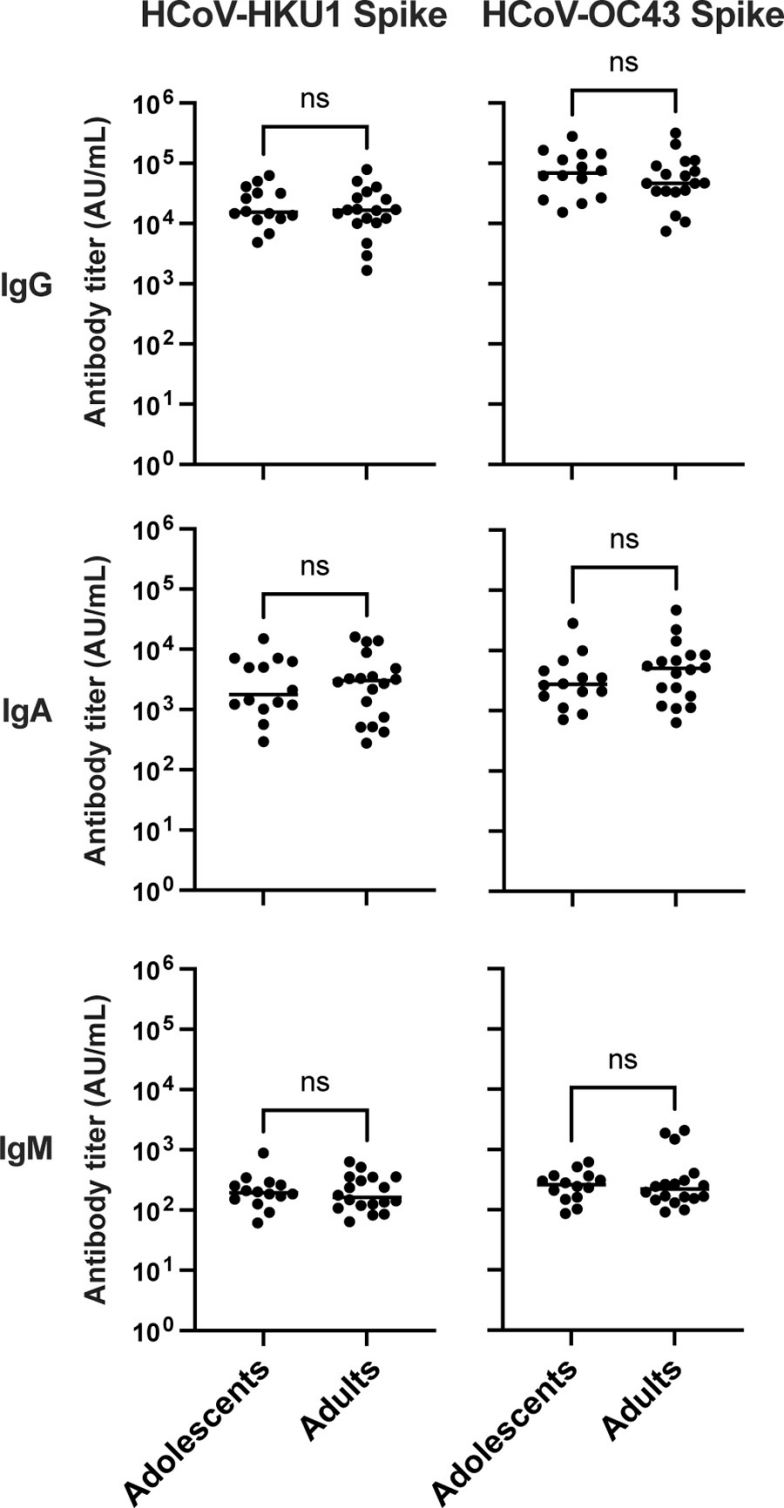
Antibody binding against seasonal beta coronavirus spike protein is not different in adult and adolescent vaccinees. Analysis of binding titers for other beta coronaviruses. Plasma samples were tested for IgG, IgA, and IgM antibody binding titers against HCoV-HKU1 Spike and HCoV-OC43 spike comparing adolescents (*n* = 15) and adults (*n* = 18) in duplicates.

**FIG 3:**
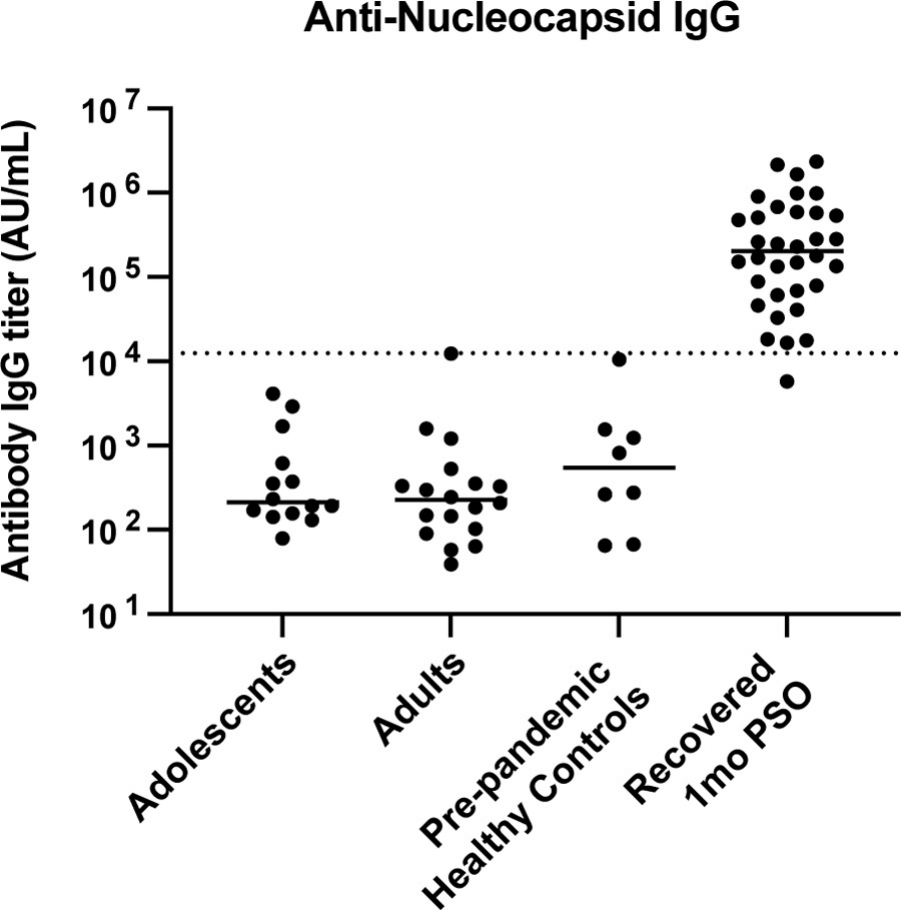
Nucleocapsid IgG binding titers are similar in adolescents and adults. Analysis of nucleocapsid IgG antibody binding titers. Adolescents (*n* = 15) and adults (*n* = 18) samples were tested. Plasma samples were run in duplicate in coronavirus MSD panel 1. None of the vaccinees scored positive compared with a control set of plasma samples, including COVID-19 patients and prepandemic samples, suggesting none of the participants had been exposed to SARS-CoV-2 infection. PSO, postsymptom onset. A comparison also showed no difference between the results from these two groups, with a *P* value of 0.4701 (nonsignificant). Dotted line represents limit of detection, which is set by nucleocapsid binding titers of prepandemic healthy controls (*n* = 8) calculating as (average +3 SD).

Importantly, we observed no difference in antibody binding titers against seasonal beta coronaviruses such as HCoV-HKU1 and HCoV-OC43 for all antibody isotypes tested between adolescents and adults (Fig. 2). Thus, this higher vaccine-induced antibody response in adolescents does not appear to be due to preexisting immunity to seasonal coronaviruses, as has been suggested (18). These participants did not have any documented history of prior SARS-CoV-2 natural infection. While previous exposure cannot be formally excluded due to declining titers, we confirmed the absence of nucleocapsid binding titers in this cohort as shown in Fig. 3.

Overall, the data presented herein shows adolescent vaccinees develop antibody responses that are moderately more potent than what is found in adults, both in terms of binding and neutralization, and that these responses are dominated by IgG antibody.

### Adolescents and adults generate humoral responses of similar breadth against a panel of VOCs.

**FIG 4:**
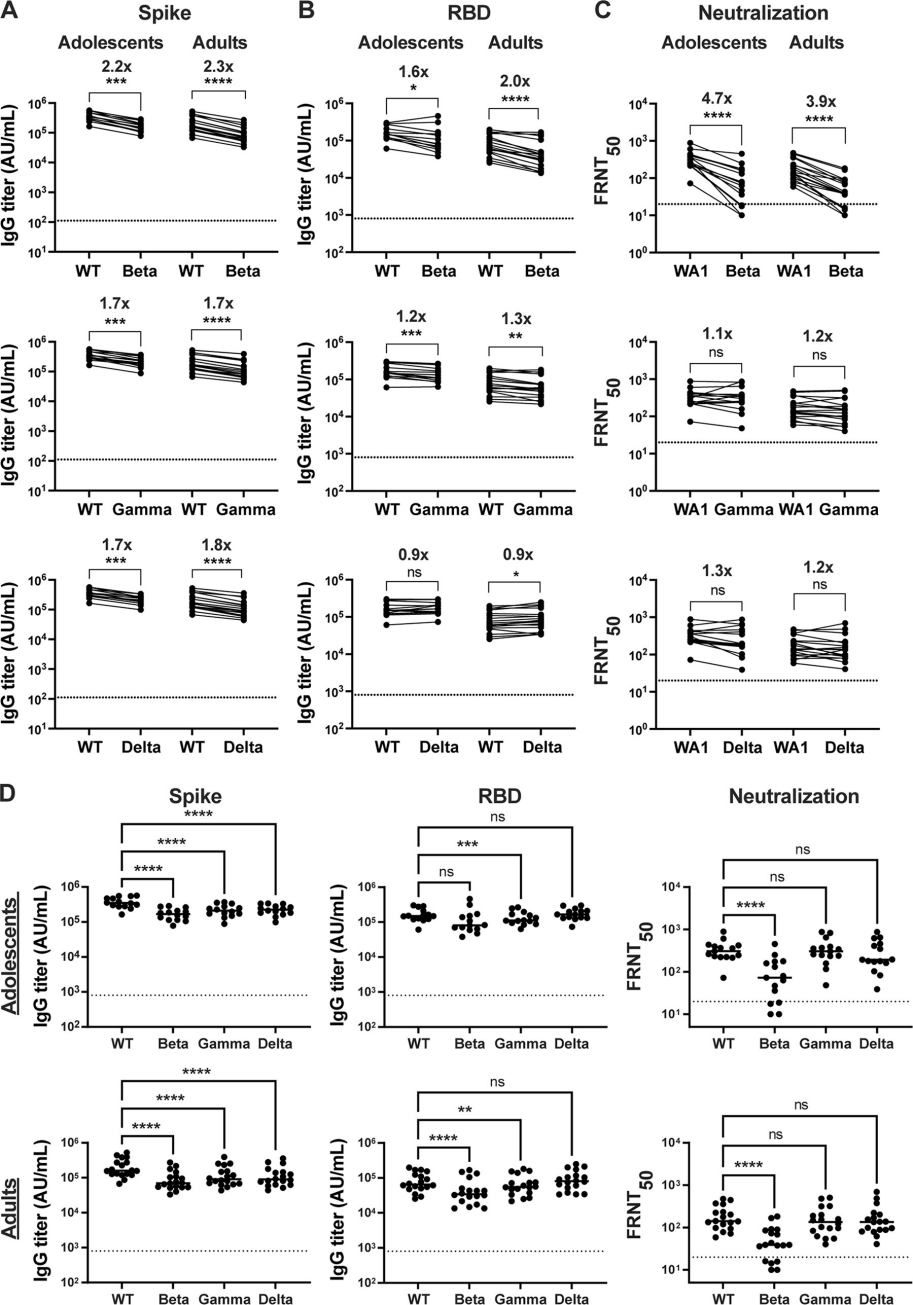
Adolescent vaccinees exhibit a similar breadth against VOCs as adults. IgG Antibody binding titers for (A) Spike and (B) RBD and (C) neutralization titers (FRNT_50_) were analyzed against VOCs, including Beta (B.1.351), Delta (B.1.617.2), and Gamma (P.1), all run in duplicate. (D) Direct comparison of titers against each virus in our cohorts shown side by side. Dotted line represents limit of detection which is set by binding titers of prepandemic adult healthy controls (*n* = 8) calculating as (average +3 SD). Both adolescent (*n* = 15) and adults (*n* = 18) plasma samples were compared. Fold change was calculated. *P* values are depicted as *, *p* < 0.05; **, *p* < 0.005; ***, *p* < 0.001; ****, *p* < 0.0001.

To address potential functional differences in the antibody repertoire, and the ability to bind and neutralize viral VOCs, we analyzed the vaccine samples herein against Beta (B.1.351), Delta (B.1.617.2), and Gamma (P.1) variants. The plasma of all vaccinated individuals retained both antibody binding for Spike (Fig. 4A), RBD (Fig. 4B), and neutralization titers (Fig. 4C) for all tested variants. In the case of Spike IgG binding titers, adolescent and adults showed a similar 2- to 3-fold reduction in binding against the Beta, as well as a 1.5- to 2-fold decline against the Delta and Gamma variants. For RBD, a 1.5- to 2-fold reduction of binding against the Beta variant was also evident, while no difference was observed against the Delta and Gamma variants. In terms of viral neutralization, we found that 3- to 5-fold reduction in titers against the Beta variant, while titers against the Delta and Gamma variants were unchanged. To allow for a direct comparison of titers against each virus in our cohorts, we also show them side by side in Fig. 4D. This clearly shows that both binding and neutralization titers are mainly affected against the Beta variant, with minor changes against Gamma and Delta variants. This is especially evident in the neutralization assays, with more modest differences in binding titers. Overall, the breadth of binding and neutralization were very similar among the adolescent and adult samples.

### Vaccinated adolescents display poor antibody potency against the emerging Omicron variant.

**FIG 5:**
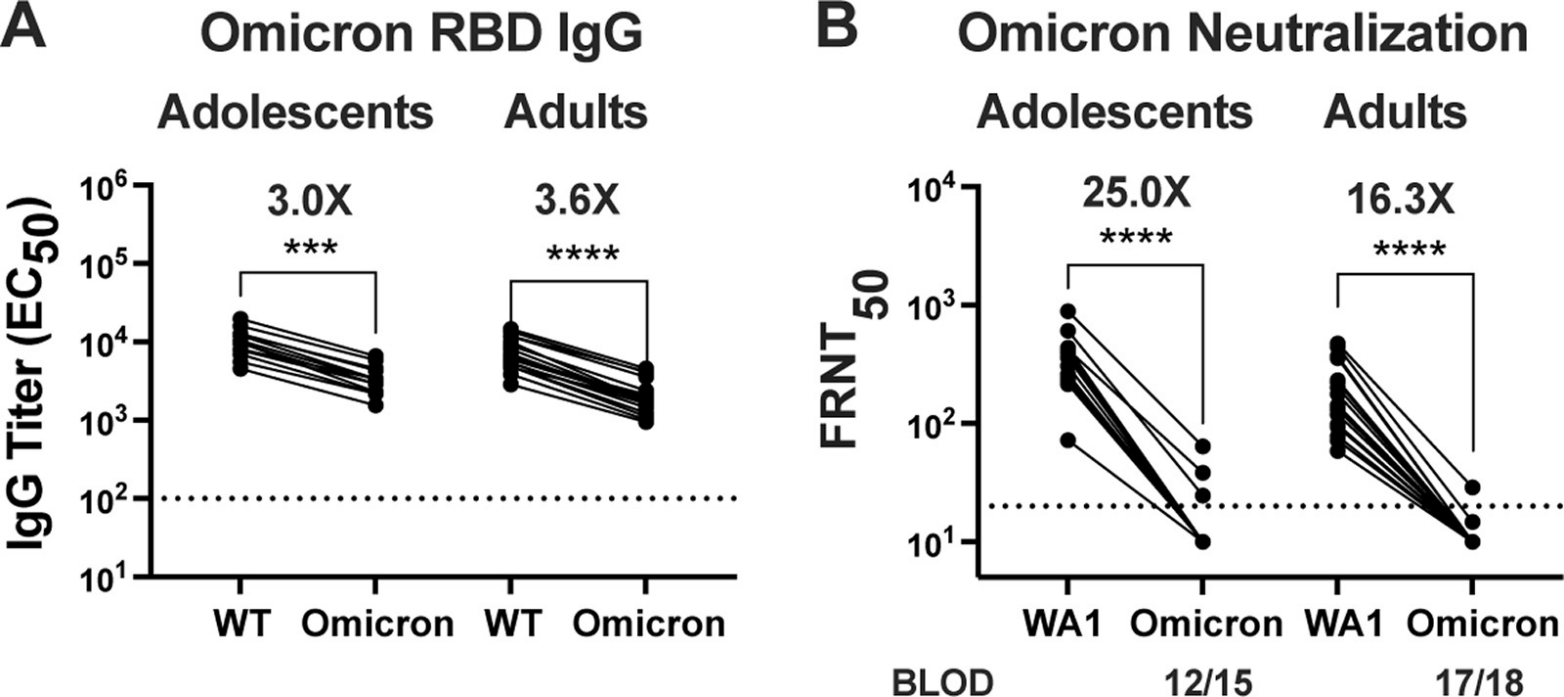
Postvaccine antibodies in adolescents and adults poorly neutralize the Omicron variant. Analysis of RBD binding and neutralization titers against wild-type and the Omicron variant. (A) RBD IgG binding titers (EC_50_) were measured and compared between wild-type and the Omicron variant. Dotted line represents minimum dilution used in ELISA (1/100). (B) Neutralization titers FRNT_50_ were measured for WA1 and Omicron variant in duplicate. Dotted line represents the limit of detection at 1/20 (with undetectable titers assigned a value of half of lowest dilution = 1/10). BLOD, below the limit of detection. Both adolescent (*n* = 15) and adults (*n* = 18) plasma samples were compared. *P* values are depicted as ***, *p* < 0.001; ****, *p* < 0.0001.

The recent emergence of the Omicron variant, and its rapid spread globally, has raised significant concern about the ability of vaccine-induced immunity to neutralize this novel variant. Similar to adults, adolescents displayed a sharp reduction in RBD binding IgG titers against the Omicron (B.1.1.529) variant compared with the wild-type RBD, as quantified by ELISA (Fig. 5A), with a 3- to 4-fold reduction in both adults and adolescents. Adolescent vaccinees showed a median IgG binding titer of 10,038 (range 4,549 to 16,000) while for Omicron it was 3,340 (range 1,541 to 6,615). In adults, the median binding titer was 6,252 (range 2,867 to 14,547) while for Omicron it was 1,787 (range 947 to 4,584). Similarly, all vaccine recipients largely failed to neutralize the Omicron variant, with detectable titers in only three of 15 adolescents and one of 18 adults (Fig. 5B). Overall, a significant reduction in the ability of vaccine induced antibody to bind, and a failure to neutralize the Omicron variant was evident in both adolescent and adult vaccinees.

## DISCUSSION

During acute SARS-CoV-2 infection, adults develop IgG, IgA, and IgM antibodies against both spike and nucleocapsid proteins, while children primarily develop spike specific IgG antibodies, with a reduced nucleocapsid response (19). This suggests children mount a response to SARS-CoV-2 infection which is different from adults in terms of magnitude, specificity, repertoire, and immunoglobulin isotype usage. However, it is not clear if this represents a fundamentally different response or simply correlates with exposure to less antigen due to a milder infection. It is also not clear how immune responses to available SARS-CoV-2 vaccines differ between adults and adolescents, especially in the context of breadth of activity against VOCs. To address this issue, we have analyzed two cohorts of adult and adolescent donors vaccinated with the Pfizer (BNT162b2) mRNA vaccine 1 month after the second dose.

The Pfizer (BNT162b2) vaccine is based on mRNA encoding the entire prefusion spike, including the RBD and the NTD. The RBD and NTD domains are key protective epitopes, with RBD being responsible for the interaction with the human ACE2 receptor, which is responsible for entry into target cells in the lung. NTD is important for interaction with L-SIGN and DC-SIGN, mediating fusion, and is an additional target for viral neutralization. Both domains are hot spots for mutations occurring in the emerging variants (20, 21). We found adolescents had higher vaccine-induced IgG binding against full-length spike and its subcomponents RBD and NTD compared with the adults. This was also the case for IgA, while no difference was observed on terms of IgM antibody. These data suggest favorable immune protection in adolescents as long-term immunity is reflected by switched antibody in both mucosal as well as systemic immune responses (22). In contrast to the reported data from naturally infected children (19), our data demonstrated moderately more potent antibody responses in adolescents compared with adults. One hypothesis for the generally mild symptoms in children, and a possible reason for the improved vaccine responses in adolescents, is that preexisting immunity against seasonal beta coronaviruses could impact both the magnitude and the repertoire of responding B cells after vaccination in adolescents. Most adults have preexisting antibodies for seasonal coronavirus such as HCoV-OC43, HCoV229E, and NL63, which increases after SARS-CoV-2 infection. However, these antibodies have not been associated with SARS-CoV-2 protection (18). We observed no difference in beta-coronavirus HCoV-HKU1 or HCoV-OC43 spike binding titers in our study between adolescents and adults that could explain the sharper increase in antibody titers in adolescents after vaccination (Fig. 2).

Neutralizing antibody responses are crucial to reduce COVID-19 disease severity (23). We observed that the neutralization activity was higher in adolescents compared with adults, similar to what was observed for IgG binding. This is in agreement with the FDA approval-enabling study by Frenck et al., demonstrating that the Pfizer mRNA vaccine induced higher neutralizing antibody responses in children, with a geometric mean ratio comparing Pfizer vaccinated adolescent (age 12 to 15) to adult (age 16 to 25) reported as 1.76 (range 1.47 to 2.10) (10).

Our data also demonstrated that a similar breadth of antibody repertoire develops in adolescents compared with adults against VOCs such as Beta, Delta, and Gamma. This provides evidence for cross-protective binding antibody responses after BNT162b2 vaccination in adolescents against VOCs with no evident difference in the neutralizing antibodies between vaccinated adolescents and adults for VOCs. With the recent emergence of the Omicron SARS-CoV-2 viral variant, children and adolescent populations are experiencing higher infection rates (8). This prompted us to also evaluate the antibody magnitude and functional breadth against the recent Omicron variant. The highly mutated Omicron variant can evade vaccine-induced immunity in adults except for vaccinees that have received a third dose, or previously infected and then vaccinated individuals (16, 24). These issues have not been fully characterized in adolescents and children. Here, we observed a marked reduction in neutralizing antibody responses to the Omicron variant. This finding underscores the potential for this VOC to evade preexisting vaccine-induced immunity and cause breakthrough disease. This is a serious concern and illustrates the urgent need for evaluating humoral immune response against Omicron infection after the third vaccine dose in adolescents, as has recently been reported for adult vaccine recipients (25).

The data presented herein is focused on humoral responses. While it would also be of interest to study cellular responses, especially given recent data highlighting the potential role of T cells in mediating durable immunity (26–28), we do not have access to peripheral blood mononuclear cells (PBMC) samples from these donors. Another limitation is the relatively small cohort size and a slight gender imbalance. As previous reports have described, females can exhibit more potent humoral immune responses than males after different infections (29, 30). It is possible that this could have impacted our findings. However, several current studies have concluded that there are minimal gender differences in COVID-19 vaccine recipients (31, 32). In addition, while not powered to address this, we found no difference in the antibody responses between male and female adolescent vaccinees. The cohort in the above study was sampled 30 days after the second dose of their initial vaccine series. It has been reported that both antibody binding titers and neutralization potential decline with time (33). Our study is limited to day 30 postvaccination, and future studies of antibody durability in this age group as well as younger children is warranted. Also, the adult samples used for this study were collected earlier in the pandemic, raising the possibility that the adolescent may have been exposed to infection in the interim. While we cannot formally exclude this possibility, the antibody titers against N protein as presented in the supplemental material suggest this is not the case.

In conclusion, adolescent vaccine recipients elicited significantly higher binding and neutralizing antibody responses to SARS-CoV-2, with an almost identical breadth against VOCs compared with adults at 1 month post-Pfizer vaccination. We also found the neutralizing activity of mRNA vaccine-induced responses in both adults and adolescents was significantly reduced against the Omicron variant, with most donors showing no detectable neutralizing activity. These findings suggest that a two-dose BNTb162b vaccine series may be insufficient to protect against the Omicron variant and support the need for ongoing evaluation of booster vaccination in adolescents.

## MATERIALS AND METHODS

### Participant sample collection.

All studies were approved by the Emory University (IRB STUDY00000723) and Washington University (IRB Study number 202012081) Institutional Review Board approvals, for adolescent and adult participants, respectively. Written informed consent and assent were obtained from all participants, as appropriate for age. The volunteers received vaccination with two doses of Pfizer (BNT162b2). Plasma samples were collected 1 month after the second vaccination. All participants were healthy at the time of enrollment with no documented prior infection with SARS-CoV-2.

### Viruses and cells.

VeroE6-TMPRSS2 cells were generated and cultured as previously described (13). VeroE6-TMPRSS2 cells were used to propagate all virus stocks. nCoV/USA_WA1/2020 (WA1), closely resembling the original Wuhan strain and the spike used in the mRNA-1273 and Pfizer BioNTech vaccine, was propagated from an infectious SARS-CoV-2 clone as previously described (34). icSARS-CoV-2 was passaged once to generate a working stock. The B.1.351 variant isolate, kindly provided by Dr. Andy Pekosz (John Hopkins University, Baltimore, MD), was propagated once to generate a working stock (12). hCoV-19/USA/PHC658/2021 (herein referred to as the B.1.617.2 variant) was derived from nasal swab collected in May 2021 (13). hCoV19/EHC_C19_2811C (herein referred to as the B.1.1.529 variant) was derived from a midturbinate nasal swab collected in December 2021. This SARS-CoV-2 genome is available under GISAID accession number EPI_ISL_7171744 (15). All the variants were plaque purified on VeroE6-TMPRSS2 cells and propagated once in a 12-well plate of confluent VeroE6-TMPRSS2 cells followed by expansion of the working stock in T175 flasks of confluent VeroE6-TMPRSS2 cells. The resulting stocks were aliquoted to generate working. All viruses used in this study were deep sequenced and confirmed as previously described (13).

### SARS-CoV-2 multiplex ECLIA immunoassay.

Plasma samples analyzed using a multiplex electro-chemiluminescent-based multiplex immunoassay provided by Mesoscale Discovery (MSD) according to the manufacturer’s instruction. The V-PLEX COVID-19 Coronavirus Panel 1 (K15362U) was used to assess wild-type (Wuhan strain) binding titers to RBD and Spike, while V-PLEX SARS-CoV-2 Panel 11 (K15455U) and V-PLEX SARS-CoV-2 Panel 19 (K15540U) were used for assessing binding to additional VOC-derived RBD and Spike, respectively. Briefly, plates were blocked with MSD Blocker A. Plasma samples were diluted 1:5,000 and plated in duplicate along with a provided Reference Standard. Then, 1× MSD SULFO-TAG Anti-Human IgG, IgA, or IgM detection antibodies were added. MSD Gold Read Buffer B was added to each plate immediately prior to reading on an MSD plate reader (MESO QuickPlex SQ 120). Data were analyzed using Discovery Workbench and GraphPad Prism (9.3.0 version) software. Plasma antibody titers in arbitrary units (AU/mL) were calculated relative to the provided Reference Standard.

### Focus reduction neutralization assay.

FRNT assays were performed as previously described (12, 13, 35). Briefly, samples were diluted at 3-fold in eight serial dilutions using DMEM in duplicates with an initial dilution of 1:10 in a total volume of 60 μL. Serially diluted samples were incubated with an equal volume of respective SARS-CoV-2 variant (100 to 200 foci per well based on the target cell). The antibody-virus mixture was then added to VeroE6-TMPRSS2 cells. Postincubation, the antibody-virus mixture was removed, and prewarmed 0.85% methylcellulose overlay was added to each well. After removing methylcellulose overlay, cells were fixed with 2% paraformaldehyde in PBS. Following fixation, permeabilization buffer (0.1% BSA, Saponin in PBS) was added to permeabilized cells. Cells were incubated with an anti-SARS-CoV spike primary antibody directly conjugated to Alexaflour-647 (CR3022-AF647). Foci were visualized on an ELISPOT reader (CTL).

### ELISA.

ELISA was performed as previously described. (36) Recombinant WT RBD (in-house) and Omicron RBD (Catalog no. 40592-V08H121; Sino Biological) were coated in 1× PBS on MaxiSorp ELISA plates at 1 μg/mL concentration. After blocking with 1% BSA, plasma samples were plated in a 4-fold dilution series beginning at 1:100. Goat anti-human IgG-HRP was used to detect antibodies (catalog no.109-036-098; Jackson Immunoresearch Laboratories). Plates were developed using *o*-phenylenediamine dihydrochloride (OPD) substrate and stopped with 1 M hydrochloric acid. Absorbance was measured at 490 nm using a spectrophotometer (SpectraMax from Molecular Device). EC_50_ was calculated using a nonlinear fit of transformed absorbance values.

### Quantification and statistical analysis.

Nonparametric *t* test analysis for antibody binding titers were performed using Mann-Whitney test. Binding and neutralization variant analysis were done by Wilcoxon matched pairs signed-rank test or repeated one-way ANOVA measures as appropriate. *, *P* < 0.05; **, *P* < 0.005; ***, *P* < 0.001; ****, *P* < 0.0001. Antibody neutralization titers were quantified by counting the number of foci for each sample using the Viridot program. The neutralization titers were calculated as 1 – (ratio of the mean number of foci in the presence of sera and foci at the highest dilution of respective sera sample). The FRNT_50_ titers were interpolated using a four-parameter nonlinear regression in GraphPad Prism 9.3.0. Samples that do not neutralize at the limit of detection at 50% are plotted at 10 and was used for geometric mean calculations.
